# From Promise to Reality: Bioengineering Strategies to Enhance the Therapeutic Potential of Extracellular Vesicles

**DOI:** 10.3390/bioengineering9110675

**Published:** 2022-11-10

**Authors:** Miguel de Almeida Fuzeta, Pedro P. Gonçalves, Ana Fernandes-Platzgummer, Joaquim M. S. Cabral, Nuno Bernardes, Cláudia L. da Silva

**Affiliations:** 1 iBB–Institute for Bioengineering and Biosciences and Department of Bioengineering, Instituto Superior Técnico, Universidade de Lisboa, Av. Rovisco Pais, 1049-001 Lisboa, Portugal; 2 Associate Laboratory i4HB–Institute for Health and Bioeconomy at Instituto Superior Técnico, Universidade de Lisboa, Av. Rovisco Pais, 1049-001 Lisboa, Portugal

**Keywords:** extracellular vesicles, exosomes, drug delivery, biogenesis, uptake, targeting, engineering, scalable production, bioreactors

## Abstract

Extracellular vesicles (EVs) have been the focus of great attention over the last decade, considering their promising application as next-generation therapeutics. EVs have emerged as relevant mediators of intercellular communication, being associated with multiple physiological processes, but also in the pathogenesis of several diseases. Given their natural ability to shuttle messages between cells, EVs have been explored both as inherent therapeutics in regenerative medicine and as drug delivery vehicles targeting multiple diseases. However, bioengineering strategies are required to harness the full potential of EVs for therapeutic use. For that purpose, a good understanding of EV biology, from their biogenesis to the way they are able to shuttle messages and establish interactions with recipient cells, is needed. Here, we review the current state-of-the-art on EV biology, complemented by representative examples of EVs roles in several pathophysiological processes, as well as the intrinsic therapeutic properties of EVs and paradigmatic strategies to produce and develop engineered EVs as next-generation drug delivery systems.

## 1. Introduction

All cells share the ability to secrete extracellular vesicles (EVs), phospholipid bilayer membrane structures enclosing a portion of their own cytoplasm [1]. These vesicles are able to transfer their cargo of biomolecules, including proteins, lipids, and nucleic acids, triggering alterations in recipient cells [2,3,4]. For this reason, EVs play essential roles in intercellular communication, being associated with multiple physiological and pathological processes.

Considering their natural ability to shuttle messages between cells, researchers and companies have shifted their attention for the promising use of EVs as natural therapeutic systems. In fact, EVs are able to mediate some of the therapeutic effects from their cells of origin [5,6]. Therefore, EVs could be used in the substitution of their cell of origin, as a cell-free therapy triggering an equivalent therapeutic effect, but avoiding the complexity and safety concerns associated with cellular therapies.

Furthermore, EVs have been explored as drug delivery vehicles for the treatment of several diseases, through the loading of therapeutic molecules and shuttling them to target cells and tissues. Their small size and resemblance to the cell membrane makes EVs ideal candidates to cross biological barriers, while offering high biocompatibility to target cells [7,8,9]. Some EVs show inherent targeting ability and display tropism towards particular cells or tissues [10,11,12]. Due to their biological origin, EVs present generally low immunogenicity and toxicity, allowing to overcome safety issues associated with synthetic nanocarriers [13,14,15,16]. Thus, EVs have emerged as promising drug delivery systems (DDS), presenting advantageous features that may allow them to outperform synthetic nanocarriers. In fact, EVs have been recently described to deliver functional RNA more efficiently than state-of-the-art synthetic RNA nanocarriers [17].

In addition to their ability to pack therapeutic molecules, EVs can be further engineered to enhance their targeting capacity towards specific tissues. This has been achieved either by the genetic engineering of parental cells in order to express a targeting moiety fused to an EV transmembrane protein, or by anchoring targeting ligands to the surface of EVs after their isolation. Here, we review recent and notable developments in the field, allowing the engineering of EVs to enhance their therapeutic potential, elucidating the pros and cons of each strategy. This is preceded by a contextualization of the biology of EVs and their roles in major pathophysiological processes. We also review the most recent strategies employed to address the critical issues in upstream and downstream processing of EVs to their scalable manufacturing. These platforms are expected to support the large numbers and high purity of EVs needed as the field moves more intensively from pre-clinical to clinical studies towards the validation of EVs as key players in therapeutic applications. 

## 2. EV Biology

The identification of EVs can be traced back to as early as 1946, when they were described as pro-coagulant particles in plasma [18], and later in the 1960s described as “platelet-dust” and as cartilage matrix vesicles associated with bone calcification [19,20]. A major breakthrough occurred in 1983, when a mechanism for the release of transferrin receptors from maturing red blood cells through vesicles was described [21,22]. These vesicles were later named “exosomes” in 1987 [23].

**Figure 1:**
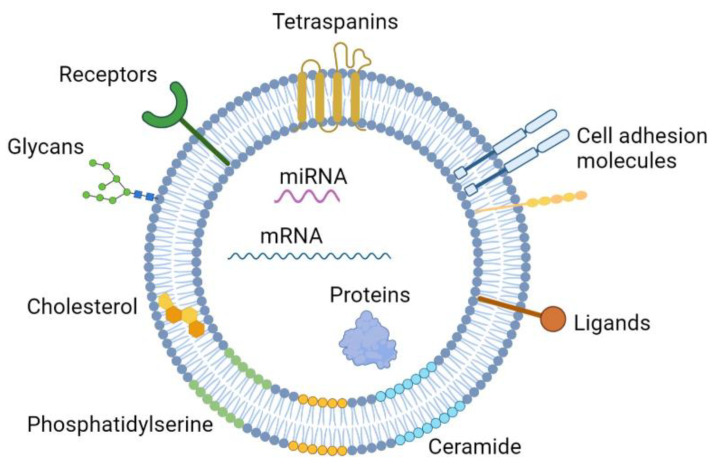
Schematic representation of an extracellular vesicle (EV) and its biological composition. EVs are composed by a phospholipid bilayer membrane enclosing intraluminal fluid with cytoplasmic origin. They contain biomolecules from their cell of origin, which include, other than lipids, several types of proteins (e.g., involved in cell adhesion, as well as other transmembrane and intraluminal proteins with various functions) and nucleic acids (e.g., mRNA and miRNA). Figure created with BioRender.com.

For some time, EVs were only considered to be a means to remove unwanted material from the cell. However, the field of EVs was revolutionized in 1996 when exosomes were shown to play a role in antigen presentation, opening an entirely new discussion that EVs might play a role in the transfer of biological information between cells [2]. This was later consolidated in 2006 and 2007, when EVs were shown to contain RNA (miRNA and mRNA) that could be delivered to recipient cells, changing their behavior [3,4]. Since then, EVs have emerged as relevant players in intercellular communication, mainly through their ability to transfer a cargo of biomolecules, including proteins, lipids, and nucleic acids, which trigger alterations on recipient cells (Figure 1).

The term EVs was proposed in 2011 to define all the different types of extracellular membrane structures [1]. However, EVs actually comprise a highly heterogeneous group. Depending on their biogenesis, EVs are broadly categorized either as exosomes, or microvesicles [9]. Exosomes are generated through the endosomal pathway [9,24]. Endocytosis at the cell membrane leads to the formation of early endosomes. During endosome maturation into late endosomes, there is an inward budding of endosomes resulting in the accumulation of intraluminal vesicles (ILV), which leads to the formation of multivesicular bodies (MVB), also named multivesicular endosomes (MVE). Upon the fusion of MVE with cell membrane, ILV are released to the extracellular space originating exosomes, which generally display 50–150 nm in diameter. Microvesicles are formed by the outward budding of the plasma membrane, ranging in size from 50 nm to 1 µm in diameter, or even higher [9]. Exosomes and microvesicles show overlapping properties, such as size, density, and molecular composition, making it challenging to distinguish different co-isolated EV subpopulations [25]. Additionally, the composition of EVs may differ among different secreting cells.

The complexity of EVs is further increased when we consider other structures, such as apoptotic bodies released from cells undergoing apoptosis, which can span over a large size range (from 100 nm to 5 µm in diameter) [25], or the recently identified mitovesicles from mitochondrial origin [26]. Interestingly, recent studies revealed that EVs could even transport whole organelles inside them, such as mitochondria, thus enabling their functional trafficking between cells [27,28]. Both the trafficking of mitovesicles, or whole mitochondria shuttled inside EVs, seem to be associated with the processes of mitochondrial regulation and dysfunction, namely in neurodegenerative disorders. Moreover, EVs display physical characteristics (i.e., size and density) similar to other secreted non-vesicular nanoparticles, such as lipoproteins of various densities [29] and the recently identified exomeres [30].

### 2.1. EV Biogenesis

Cargo incorporated in exosomes originate from endocytosis at the plasma membrane or are directly targeted to early endosomes via the biosynthetic pathway, from the *trans*-Golgi network [9]. These sorting processes are regulated by various Rab GTPases. The formation of ILV can be regulated by the endosomal sorting complex required for transport (ESCRT), a family of proteins that associate in a stepwise manner at the membrane of MVE [9,25]. Firstly, ESCRT-0 and ESCRT-I subunits cluster membrane-associated cargo in microdomains of the limiting membrane of MVE. The tumor susceptibility gene 101 protein (TSG101) is one of the main ESCRT-I components, being used as an EV protein marker. This is followed by ESCRT-II-mediated recruitment of ESCRT-III that performs budding and fission of this microdomain into the MVE lumen.

Although ESCRT-III is required for fission of ILVs, cargo clustering and membrane budding can be either ESCRT-dependent or ESCRT-independent [9]. The latter can rely on syntenin and the ESCRT accessory protein ALG-2 interacting protein X (ALIX), which links cargo to ESCRT-III [9,31]. ESCRT-independent biogenesis is aided by lipids such as ceramide, which allows the generation of membrane subdomains imposing a spontaneous curvature on the membranes [32,33]. Additionally, proteins of the tetraspanin family (e.g., CD63, CD81 and CD9) have been shown to regulate ESCRT-independent cargo sorting to exosomes [9,34,35]. Some tetraspanins also show the potential to form microdomains and induce budding.

Mature MVE can follow a degradative route by fusion with lysosomes or autophagosomes. Alternatively, MVE are transported along microtubules to the plasma membrane. At this stage, MVE fuse with the plasma membrane leading to exosome release in a process mediated by Rab GTPases (e.g., Rab27A/B, Rab35), actin and SNARE (soluble *N*-ethylmaleimide-sensitive fusion attachment protein receptor) proteins [9,25,36,37,38].

Microvesicle biogenesis shares several mechanisms common to exosome biogenesis. This includes the formation of microdomains (in this case in the plasma membrane) where specific lipids and cargo are clustered, as well as a similar role of ESCRT machinery and ceramide in vesicle formation [9]. However, microdomain formation is followed by the translocation of lipids between leaflets of the plasma membrane, a process unique to microvesicle formation. This process is mediated by Ca^2+^-dependent enzymes (e.g., translocases, scramblases and calpain), rearranging the asymmetry of membrane phospholipids in a way that causes the physical bending of the membrane, favoring membrane budding [25,39]. The most significant examples are the exposition of phosphatidylserine (PS) and phosphatidylethanolamine (PE) from the inner leaflet to the cell surface.

### 2.2. EV Interaction with Recipient Cells

After being released into the extracellular space, EVs are able to interact with cells either close-by or far away, triggering phenotypic changes in these cells. EV binding to recipient cells can be mediated by tetraspanins, integrins, proteoglycans, lectins, lipids (e.g., PS) and extracellular matrix components (e.g., fibronectin and laminin) [9]. After binding to a recipient cell, EVs can follow multiple routes to deliver their message. EVs can elicit changes in recipient cells by simply binding to specific surface receptors, triggering signaling pathways (e.g., antigen presentation), but without delivering any EV cargo [9].

**Figure 2:**
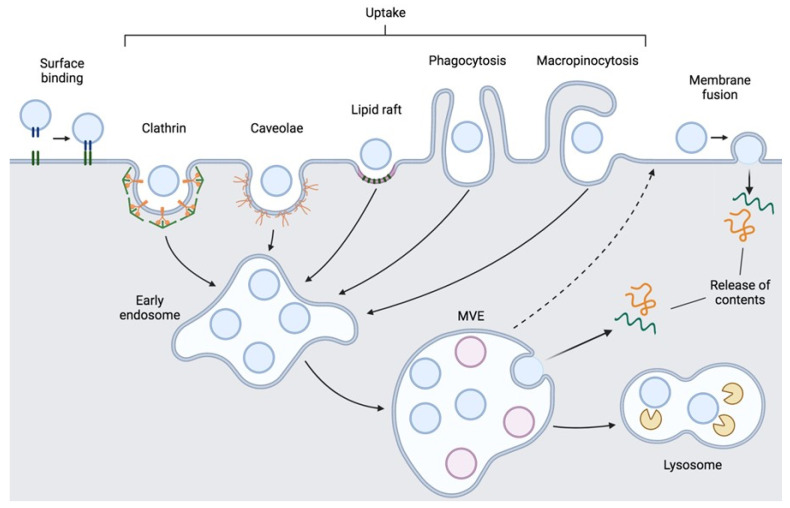
Interaction of extracellular vesicles (EVs) with recipient cells. EVs bind to the surface of recipient cells in a process that can be mediated by several molecules, being able to elicit functional changes without entering the cell. EVs may also be internalized by recipient cells through different uptake routes, which include clathrin- or caveolin-mediated endocytosis, endocytosis mediated by lipid rafts, phagocytosis or macropinocytosis. Internalized vesicles follow the endosomal pathway, being sorted into early endosomes and proceeding to MVE. Then, EVs can follow different routes: they can be recycled back to the plasma membrane and released; EVs can fuse with the limiting membrane of MVE releasing their contents to the cytoplasm of the recipient cell; or MVE may fuse with lysosomes leading to EV degradation. In alternative to EV uptake, EVs may also fuse directly with the plasma membrane of the recipient cell, releasing their cargo directly into the cytosol. MVE—multivesicular endosomes. Figure created with BioRender.com.

EVs can also be internalized through multiple EV uptake pathways [40] (Figure 2). EVs can undergo clathrin-mediated endocytosis, through the formation of a clathrin coat in a portion of cell membrane surrounding the EV to be internalized [41]. This clathrin coat promotes membrane deformation, which results in membrane invagination and formation of a bud that surrounds the EV that then pinches off, separating itself from the membrane [42]. Once in the cytosol, this internalized vesicle undergoes clathrin un-coating. EVs can also be internalized by clathrin-independent endocytosis, such as caveolin-mediated endocytosis, involving the formation of cave-like invaginations in the plasma membrane named caveolae, which become internalized into the cell (similarly to clathrin-mediated endocytosis) [40,43]. Caveolin-1 is required for the formation of caveolae, which are also rich in cholesterol and sphingolipids. Clathrin-independent endocytosis may also occur via lipid rafts [44]. These plasma membrane microdomains have altered phospholipid composition, being more tightly packed and consequently less fluid, but float freely in the plasma membrane. Lipid rafts can be found in invaginations formed by caveolin-1 or in planar regions of the plasma membrane associated with flotillins [40]. However, lipid raft-mediated endocytosis of EVs seems to be caveolae-independent [41,44].

Alternatively, EV uptake can happen through non-specific processes such as phagocytosis and macropinocytosis [40,45,46]. Phagocytosis involves the formation of invaginations surrounding material to be internalized, with or without the formation of enveloping membrane extensions [47]. Although this process is generally used to internalize larger particles, such as cells, it has been observed to be used to take up EVs. Phosphatidylinositol-3-kinase (PI3K) plays an important role in this process [48]. In macropinocytosis, membrane extensions are formed surrounding a portion of extracellular fluid and fuse back with the plasma membrane internalizing that portion of extracellular content [40,47]. This requires Na^+^/H^+^ exchanger activity and is dependent on actin, cholesterol and the rac 1 GTPase. Both of these uptake mechanisms seem to be triggered (at least partially) by PS present on the outer leaflet of EV membranes.

Internalized EVs follow the endosomal pathway, eventually reaching MVE. At this stage, EVs can follow different fates [9,25]. They can be recycled back to the plasma membrane and released to the extracellular space. MVE can fuse with the lysosome leading to the degradation of the contents of internalized EVs, which can still be a relevant source of metabolites for the host cell. Alternatively, EVs may undergo endosomal escape, through back fusion with the limiting membrane of MVE, releasing their contents to the cytoplasm of the recipient cell. EVs may also fuse directly with the plasma membrane, releasing their cargo directly into the cytosol of the recipient cell. Intraluminal material released by internalized EVs includes nucleic acids (miRNA, mRNA), proteins and lipids, which are able to trigger alterations in the recipient cell. However, little is known about how EV cargo is unpackaged and delivered to the designated site of action, either the cytoplasm or the nucleus. Intracellular delivery routes are being investigated and may include direct transfer into the endoplasmic reticulum [49] or the nucleus [50,51].

## 3. EVs in Intercellular Communication

### 3.1. Physiological Roles of EVs

Given their ability to elicit changes in recipient cells, EVs have been implicated in numerous physiological processes [52,53]. In fertilization, EVs secreted from the egg promote sperm-egg fusion in a tetraspanin CD9-dependent process that was observed in mice [54]. Later, microvesicles released by early embryo cells promote trophoblast migration and implantation in the uterus through the JNK and FAK pathways, which are activated by microvesicle cargo proteins laminin and fibronectin [55]. EVs have been implicated in development by carrying key morphogen molecules, such as Wnt proteins (e.g., Wingless) and Sonic Hedgehog [56,57,58]. Mating behavior can also be altered by EVs, since exosomes secreted by the reproductive glands of male *Drosophila melanogaster* interact with female reproductive tract epithelium and inhibit the re-mating of females [59].

EVs are important in the nervous system, being secreted by neurons and glial cells alike to mediate intercellular communication [60]. Neuron-derived EVs have multiple relevant roles at synapses, such as promoting synaptic growth at the neuromuscular junction and regulating postsynaptic retrograde signaling [61,62,63]. Oligodendrocyte-derived EVs are able to promote neuronal viability and increase neuron firing rate [64]. EVs also play a role in the peripheral nervous system, where Schwann cells are able to secrete EVs to promote axon regeneration [65].

EVs play a relevant part in the regulation of immune responses through exchange among multiple types of immune cells. EVs play a crucial role in major histocompatibility complex (MHC) class II antigen presentation, since dendritic cells (DC) secrete exosomes carrying peptide-containing MHCII that stimulate naïve CD4^+^ T cells [66,67]. DC-derived exosomes were also able to differentiate T helper cells toward a T helper type 1 (Th1) phenotype and enhance immunogenicity in vivo [68]. In the opposite direction, T cell-derived exosomes are able to transport miRNA to antigen-presenting cells, modulating their mRNA expression levels [69]. Exosomes were also found to transfer miRNA between DC in vivo, modulating gene expression in the recipient cell [70]. Another study revealed that exosome-mediated miRNA transfer from T regulatory (Treg) cells to Th1 cells was able to reduce inflammatory responses of recipient Th1 cells [71].

Physiological tissue regeneration processes are also supported by EVs. Endothelial cell-derived EVs were able to reduce atherosclerotic lesion formation when delivered to smooth muscle cells [72]. The authors of this study observed that alterations in endothelial cells, which were previously described to be triggered by blood flow-induced shear stress, led to the enrichment of endothelial cell-derived EVs in specific miRNA molecules that had atheroprotective effects after delivery to smooth muscle cells. In a kidney injury model, injured epithelial cells secreted exosomes that activated fibroblasts to initiate tissue regenerative responses and fibrosis mediated by exosomal transforming growth factor (TGF)-β1 mRNA [73].

### 3.2. EVs in Pathological Processes

In addition to their relevant role under normal physiological conditions, EVs have been associated with multiple pathological processes [52,53,74]. Numerous studies reveal tumor-derived EVs as relevant mediators of intercellular communication within the tumor microenvironment (TME), which is composed of multiple non-tumorigenic cells able to collectively support tumor growth and progression, such as endothelial cells, fibroblasts, and immune cells, among others [75].

Under hypoxic conditions (1% O_2_), glioblastoma multiforme (GBM)-derived exosomes amplified the activation of ERK1/2 MAPK, PI3K/Akt and FAK pathways in endothelial cells, compared to normoxic conditions, resulting in increased endothelial cell sprouting [76]. GBM-derived EVs were also found to skew monocyte-to-macrophage differentiation to a tumor-supportive M2-type macrophage phenotype [77]. Conversely, lymph node macrophages were able to suppress tumor growth by absorbing tumor-derived EVs and preventing their interaction with pro-tumorigenic B cells [78]. Tumor-derived EVs are also able to suppress anti-tumor adaptive immunity. Tumor-derived EVs induced apoptosis of CD8^+^ T cells and were also able to alter the differentiation of CD4^+^ T cells into a state that suppresses cytotoxic T cell activity, contributing to tumor escape from the immune system [79]. 

Tumor-derived EVs also promote tumor invasion and metastasis. They help establishing pre-metastatic niches, by interacting with normal cells at the metastatic sites. Melanoma-derived exosomes recruited bone marrow progenitor cells to future sites of metastasis and re-educated them toward a vasculogenesis supporting phenotype, enhancing tumor invasion and metastasis in vivo [80]. This re-education effect was mediated by a tyrosine kinase receptor differentially expressed in exosomes from highly metastatic melanoma cells compared to less aggressive ones.

In another study, exosomes from pancreatic cancer cells induced the formation of pre-metastatic niche in the liver of mice [81]. These exosomes transferred migration inhibitory factor (MIF) to Kupffer cells in the liver that secreted TGF-β, which subsequently increased fibronectin production by hepatic stellate cells. Fibronectin enhanced the recruitment and retention of bone marrow-derived macrophages, establishing an environment favorable for metastasis [81].

Remarkably, the formation of pre-metastatic niches was found to be promoted by exosomes from different tumor types that targeted specific organs, depending on integrins displayed on their membrane [82]. Exosomes expressing integrins α_6_β_4_ and α_6_β_1_ were able to bind specifically to fibroblasts and epithelial cells in the lung, mediating lung tropism, while exosomes containing integrin α_v_β_5_ bound to Kupffer cells, leading to liver tropism. 

EVs derived from non-tumorigenic cells can also support tumor growth under specific circumstances. For instance, exosomes from astrocytes were found to support tumor growth in brain metastatic breast cancer in vivo. Astrocyte-derived exosomes mediated miRNA transfer to metastatic tumor cells, reducing the levels of a target mRNA encoding for the tumor suppressor PTEN [83]. Decreased PTEN levels triggered and increased secretion of CCL2 chemokine by metastatic tumor cells, resulting in recruitment of IBA1-expressing myeloid cells that enhanced proliferation and reduced apoptosis of metastatic tumor cells.

In addition to their roles as mediators in tumor progression, tumor-derived EVs also provide a way to eliminate chemotherapeutic agents from cancer cells, enabling chemotherapy resistance. The microvesicle-mediated release of gemcitabine was identified as a key factor for resistance to this drug in human pancreatic cancer cells, both in vitro and in vivo [84]. Moreover, just as tumor-stromal interactions mediated by EVs play a relevant role in tumor progression, stromal cell-derived EVs can also mediate resistance to therapy. Exosomes derived from stromal cells were able to mediate miRNA transfer to ovarian cancer cells, increasing their chemoresistance to paclitaxel [85].

EVs are also involved in the cell-cell transport of pathogenic proteins associated with neurodegenerative diseases, such as the prion protein (PrP) abnormal isoform PrP^SC^ in prion disease, β-amyloid in Alzheimer’s disease and α-synuclein in Parkinson’s disease [86,87]. However, the relevance of EV-mediated versus EV-independent spread and propagation of these proteins in disease progression is still unclear. Contrastingly, some studies have described the natural beneficial effects of EVs in these pathologies, namely in the clearance of β-amyloid peptides [88,89].

In cardiovascular diseases, EVs have also been found to mediate the cross-talk between different cell types in the heart, with implications in disease progression. The secretion of cardiac fibroblast-derived exosomes triggered gene expression alterations in cardiomyocytes, leading to increased pathological cardiac hypertrophy, which contributes to heart failure [90]. In another study, macrophage-derived exosomes transferred miRNA to cardiac fibroblasts, suppressing fibroblast proliferation and promoting fibroblast inflammation during cardiac injury in mice [91].

In multiple infectious diseases, viruses are able to take advantage of EVs to transfer their genetic material between infected and non-infected cells in the substitution of direct interaction between viruses and target cells [92]. For example, exosomes derived from human hepatoma cells infected with hepatitis C virus were able to transmit the infection to naïve hepatoma cells [93]. Moreover, this exosome-mediated transmission was partially resistant to antibody neutralization.

Considering the numerous roles of EVs in disease progression, EVs have been extensively studied as novel biomarkers for disease [94,95,96], as well as targets for new therapeutic strategies [97,98].

## 4. EVs as Reconfigurable Natural Therapeutic Systems

### 4.1. EVs as Intrinsically Therapeutic Agents

Given their ability to participate in intercellular communication, conveying messages from their cells of origin to target recipient cells, EVs have innate therapeutic potential, particularly interesting for tissue regeneration. EVs are able to mediate some of the therapeutic effects from their cells of origin by carrying lipids, proteins, and genetic material (mRNA and miRNA), and transferring this cargo to target cells, or by triggering signaling pathways through cell surface interactions.

EVs derived from stem and progenitor cells have gained particular interest due to numerous therapeutic properties attributed to them, which include: immunomodulatory capacity (mainly by reducing inflammation) [99,100,101,102]; suppressing apoptosis and stimulating cell proliferation [103,104]; promoting angiogenesis [105,106]; stimulating wound repair [107,108]; and recruiting and reprograming cells for tissue regeneration [3]. Among the most studied EV-secreting cells with therapeutic properties we can find mesenchymal stromal cells (MSC), embryonic stem cells, induced pluripotent stem cells (iPSC), cardiac progenitor cells, and DC [109].

In particular, a growing body of evidence indicates that many of the therapeutic features of MSC, currently under study in numerous clinical trials, are exerted in a paracrine manner and mediated by EVs. The paracrine activity of MSC was initially observed in mice and pig models of myocardial infarction, where conditioned medium from MSC cultures limited infarct size and improved heart function [110,111,112,113]. This was followed by similar evidence supporting the paracrine activity of MSC in other organs [114]. In subsequent studies, EVs secreted by MSC were described as the mediators of these paracrine trophic activities, reducing myocardial ischemia/reperfusion injury [5] and also allowing improved recovery from acute kidney injury [6,115] in mice.

Numerous studies followed, reporting the different therapeutic activities of EVs derived from MSC and other cells. MSC-derived EVs (MSC-EVs) allowed improved recovery from stroke in mice, by promoting neuronal survival and angioneurogenesis [116]. Human bone marrow MSC-EVs allowed a better recovery from traumatic brain injury in mice [117] and improved the recovery from acute spinal cord injury in rats [100]. Attenuated inflammation upon EV treatment supported a better recovery in both studies.

In the context of MSC-EVs, vesicles obtained from different tissue sources showed therapeutic potential against hepatic indications. Human umbilical cord matrix MSC-EVs ameliorated liver fibrosis in mice by inactivating the TGF-β1/Smad signaling pathway, and inhibiting epithelial-to-mesenchymal transition (EMT) in hepatocytes [118]. Bone marrow MSC-EVs reduced hepatic injury in mice, improving their survival [119]. Reduction in hepatocyte apoptosis was proposed to be mediated by the lncRNA Y-RNA-1 carried by EVs. Human umbilical cord matrix MSC-EVs also inhibited pulmonary infiltration of macrophages and suppressed the production of pro-inflammatory and pro-proliferative factors in a murine model of pulmonary hypertension [99].

The large number of preclinical studies using EVs has already been translated into a few clinical trials. The safety and efficacy of MSC-EVs have been evaluated in clinical trials for the treatment of type 1 diabetes (NCT02138331), macular holes (NCT03437759) and chronic kidney disease, with positive safety and efficacy results in the latter [120].

More recently, MSC-EVs have been proposed for the treatment of coronavirus disease-19 (COVID-19), aiming to reduce dysregulated immune responses and the cytokine storm associated with respiratory pathological states of this disease [121]. The rationale for using MSC-EVs is based on previously mentioned observations of inflammatory attenuation in several pathological conditions, and supported by studies in relevant lung disease models, including lung injury [122,123]. However, the mechanisms behind the beneficial effects of EVs are not fully elucidated yet. Some phase I/II and even phase III clinical trials have already been registered in different countries for the use of EVs for the treatment of COVID-19, most of them using EVs derived from MSC either administered intravenously (e.g., NCT04798716, NCT05354141) or by inhalation (e.g., NCT04602442, NCT04276987) (clinicaltrials.gov, accessed on 15 September 2022 using the search term “(extracellular vesicles OR exosomes) AND COVID-19”).

### 4.2. EVs as Drug Delivery Systems

In addition to their use as innate therapeutic products mainly in the context of regenerative medicine, EVs are also promising vehicles for drug delivery to treat numerous conditions. Given their small size and the ability to shuttle messages to other cells in virtually any site in the organism eliciting a functional response, EVs can be regarded as nature’s nanocarriers. In fact, EVs comprise numerous features that make them appealing for the development of novel DDS, even outperforming synthetic nanocarriers (e.g., lipid nanoparticles [17]) or viral delivery platforms (e.g., adeno-associated virus (AAVs) [124]) in certain aspects.

By using EVs, we can take advantage of endogenous cellular machinery to produce the desired therapeutic cargo and sorting it inside EVs. Additionally, EVs have the ability to overcome biological barriers, namely tissue barriers (e.g., blood-brain barrier (BBB)), cellular barriers (by different EV uptake mechanisms) and intracellular barriers, exerting functional effects on target cells [7,125,126]. Due to their biological origin, EVs are generally low immunogenic and are not toxic, contrarily to some synthetic nanocarriers, with different studies revealing minimal hepatoxicity and overall toxicity of human-derived EVs both in vitro and in mouse models [13,15,127,128]. On the other hand, EVs are non-replicative and non-mutagenic, relieving some of the safety concerns associated with cell therapies. Therefore, EVs lie in a sweet spot between synthetic nanocarriers and cell therapies, presenting exciting opportunities for developing next-generation DDS with increased efficacy and lower side-effects.

EVs are able to carry different cargos, including small molecules such as the natural compound curcumin or chemotherapeutic drugs (e.g., doxorubicin and paclitaxel), as well as delivering proteins and different RNA molecules (e.g., siRNA, miRNA, and mRNA) [7,129,130,131,132,133]. In this way, EVs can deliver therapeutic molecules in a more efficient and selective manner to target diseased cells and tissues, while minimizing their side effects, as well as protecting cargo from degradation (particularly relevant for RNA molecules). For example, doxorubicin-loaded EVs showed similar cytotoxicity to the free drug in both in vitro and in vivo models of breast cancer, but with reduced cardiotoxicity [130].

Therapeutic cargo can be loaded into EVs by two different strategies, either exogenously, by inserting cargo directly into EVs after EV production and isolation, or endogenously, where therapeutic cargo is loaded into EVs at the moment of EV biogenesis [126,134]. Several techniques have been applied in order to accomplish the exogenous loading of EVs including direct incubation, electroporation, sonication, saponin, freeze/thaw cycles, or extrusion [134]. For example, curcumin was loaded into EVs through direct incubation (e.g., mixing at 22 °C for 5 min) in several studies, yielding diverse positive therapeutic outcomes such as improved bioavailability and anti-inflammatory effect of this drug in a mouse model of inflammation [129], as well as enhanced tumor growth inhibition both in vitro and in vivo, compared to free curcumin [135]. Electroporation has been applied in numerous studies, for example to load EVs with therapeutic siRNA with positive outcomes in mouse models of Alzheimer’s disease [7] and pancreatic ductal adenocarcinoma (PDAC) [14], or for loading small molecules such as doxorubicin, with improved outcomes in different in vivo cancer models [130,136,137].

A few studies compared the efficiency of the exogenous loading of EVs using different techniques. The hydrophobicity of small molecules can influence their loading into EVs, since hydrophobic porphyrins were loaded in EVs from different cells simply by direct incubation, while hydrophilic porphyrins benefited significantly from active loading techniques such as electroporation, extrusion, and especially saponin treatment [138]. In another study, exosomes were loaded with catalase and tested in in vitro and in vivo models of Parkinson’s disease [139]. Different loading techniques were tested (incubation, saponin treatment, freeze/thaw cycles, sonication, and extrusion), revealing improved loading efficiency, sustained release, and catalase preservation upon active loading, especially using sonication, extrusion, or saponin treatment. Sonication also improved exosome loading with paclitaxel and yielded positive therapeutic outcome in multidrug resistant (MDR) cancer cells [131]. However, active loading techniques induce the temporary disruption of the EV membrane that can lead to a loss of EV content, or altered morphology, and may also induce the aggregation of EVs or their cargo, as previously reported [140,141].

Additionally, EVs can be fused with liposomes previously synthesized to carry therapeutic cargo, thereby creating EV-liposome hybrids carrying this cargo [142]. MSC-EVs fused with liposomes loaded with a chemotherapeutic compound increased the drug delivery efficiency when compared with the free drug or the drug-loaded liposome in cancer in vitro models [143]. EVs may even be combined with biomaterials to improve their functional delivery to target tissues. For example, the injection of hydrogels of multiple formulations carrying EVs allowed sustained EV release over time, enhanced local EV retention and improved therapeutic outcomes in different pathological models, both in vitro and in vivo [144].

The endogenous loading of EVs can be achieved by taking advantage of the natural sorting machinery of cells for the production and/or loading of cargo into EVs. Cells can be loaded with a cargo by direct incubation, which is then sorted and released inside EVs. MSC incubated with paclitaxel incorporated this chemotherapeutic agent and released it inside EVs [145]. These paclitaxel-loaded MSC-EVs were able to inhibit tumor cell proliferation in vitro.

Alternatively, endogenous loading of EVs can be achieved by the genetic modification of parental cells to express desired RNA molecules or proteins, which will then be loaded into EVs. For example, adipose tissue-derived MSC were genetically modified to express miR-122, previously reported to reduce drug resistance in hepatocellular carcinoma [146]. Modified MSC secreted EVs packaging miR-122, which increased antitumor efficacy of chemotherapy on hepatocellular carcinoma in vivo.

A new system was developed to achieve protein loading into EVs using an optically reversible protein-protein interaction module [147]. The authors used a photoreceptor cryptochrome 2 (CRY2) and the CRY-interacting protein (CIBN), which bind under blue light illumination. CRY2 was fused with a cargo protein and CIBN was conjugated with the EV transmembrane protein CD9. As a result, the transient docking of CRY2-conjugated cargo proteins with CD9-conjugated CIBN was observed in the generated exosomes in the presence of blue light. When the blue light was removed, the proteins detached and the cargos were released into the intraluminal space of exosomes, allowing cargo proteins to be delivered to recipient cells both in vitro and in vivo. This strategy was used for the delivery of super-repressor IκBα to relieve sepsis-associated organ damage and reducing mortality in mice [148].

In addition to their unique drug loading abilities, EVs also exhibit intrinsic targeting properties that can be valuable for drug delivery, since the protein and lipid composition of EVs can influence cell/organ tropism [12]. As previously mentioned, depending on the integrins displayed on their membrane, EVs can show tropism towards lung or liver in pre-metastatic niche formation [10]. Another example is the involvement of PS in EV recognition and uptake by macrophages [11].

Still, EVs can be engineered in order to improve specificity to target cells. Akin to the techniques used for endogenous drug loading, parental cells can be genetically modified to express a targeting moiety fused to an EV transmembrane protein. The first example relied on the fusion of lysosome-associated membrane protein 2 (Lamp2b), abundant on the surface of EVs, with the rabies viral glycoprotein (RVG) peptide that binds to the acetylcholine receptor [7,149]. In the context of Alzheimer’s disease, this strategy allowed EVs to target neurons, oligodendrocytes, and microglia, and functionally deliver electroporated siRNA to the brain in mice. The fusion of targeting moieties with Lamp2b has been used to endow EVs with targeting capacity in several studies, including an α_v_ integrin-targeting iRGD peptide to target tumor cells and tumor-associated vascular endothelium for doxorubicin delivery [150], and a fragment of interleukin 3 (IL3) to target IL3 receptors on chronic myeloid leukemia (CML) cells for delivery of a chemotherapeutic agent or siRNA [151]. 

Different types of targeting moieties and transmembrane proteins have been used (Table 1). For example, EV-producing cells have been modified to express recombinant anti-epidermal growth factor receptor (EGFR) nanobodies fused to glycosylphosphatidylinositol (GPI)-anchoring peptides [152]. Since EVs are enriched in GPI, EVs were enriched in GPI linked nanobodies displayed on their surface. This allowed EVs to target specifically EGFR-expressing tumor cells.

Alternatively, targeting ligands can be exposed on the surface of EVs after EV isolation, avoiding the challenging genetic engineering of producer cells. Several different strategies have been applied in this context (Table 2). A recombinant protein was developed, consisting of an anti-EGFR nanobody fused to the C1C2 domain of lactadherin, which binds to PS present on the surface of EVs, directing these modified EVs to EGFR-positive cancer cells [156]. In another study, EVs were modified in order to target neuropilin-1 (NRP-1), which is a transmembrane glycoprotein overexpressed in glioma cells and the tumor vascular endothelium [157]. For this purpose, an NRP-1-targeting peptide was conjugated with the surface of EVs by click chemistry.

The promising results obtained in preclinical studies using EV-based DDS for numerous clinical indications, prompted their use in pioneering clinical trials over the past few years [109]. Plant-derived EVs loaded with curcumin have been used for the treatment of colon cancer (NCT01294072). Tumor-derived EVs loaded with chemotherapeutic agents (NCT01854866) and EVs derived from malignant pleural effusion loaded with methotrexate (NCT02657460) are being studied for the treatment of malignant ascites and pleural effusion. Other clinical trials are testing the use of allogeneic MSC-EVs enriched in miR-124 for the treatment of acute ischemic stroke (NCT03384433), or loaded with KRAS^G12D^ siRNA for the treatment of metastatic pancreatic cancer (NCT03608631).

## 5. Production and Isolation of EVs

### 5.1. Upstream Processing

One of the main challenges faced in the field of EVs is the low yield obtained during manufacturing [166]. This renders basic scientific research, pre-clinical studies, and clinical trials more challenging, leading to a significant delay in the progress of the field in order to harness the full potential of EVs in clinical settings [167]. The whole bioprocessing pipeline, from upstream to downstream processing, needs to be considered for EV manufacturing optimization. Upstream processing comprises cell culture leading to EV production, thus requiring the appropriate choice of both the cell culture medium used and the culture vessel, which can range from small vessels such as T-flasks up to 500 L bioreactors, depending on the chosen approach. This step influences the characteristics of the final product and therefore should be carefully selected, while having in mind a specific application.

Progress is already being made towards optimizing the upstream conditions of EV production through the adaptation of already well-established technologies in other fields to produce functional and safe EVs in a cost-effective way. The scaling-up or scaling-out of such platforms is expected to decrease the costs associated with EV manufacturing [168]. The simplest scaling option is to adopt the use of multi-layer flasks (e.g., hyperflasks, Cellstacks, Cell Factories) instead of the use of conventional T-flasks [168]. These systems present several layers for cell growth, decreasing the manipulation time needed, in comparison to conventional T-flasks and reducing lab footprint. For example, hyperflasks were used for the cultivation of bone marrow-derived MSC and the EVs isolated from the conditioned medium (i.e., containing EVs) were shown to significantly suppress the symptoms of graft vs. host disease (GvHD) in a murine model [169]. However, culture monitoring is challenging when using these planar systems, which do not lead to cost savings in the whole process, namely in what concerns the quantity of cell culture medium used [170].

Bioreactors represent promising scale-up or scale-out options for EV manufacturing, which, under optimized conditions, can generate higher cell and EV yields in a more cost-effective way (e.g., lower need for consumables such as culture medium), compared to static systems [168]. In fact, some bioreactor systems were already explored for EV production.

Hollow-fiber bioreactors allow adherent cells to grow attached to the fibers composed of a permeable membrane, while the cell culture medium flows across the membrane. In a recent study, it was observed that umbilical cord matrix MSC-EVs produced in a hollow-fiber system displayed enhanced osteochondral regeneration activity when compared to 2D culture flasks, leading to superior repair activity of cartilage defects in vivo [170]. Another study revealed that umbilical cord matrix-derived MSC expanded in hollow-fiber bioreactors produced EVs with enhanced renoprotective effects in comparison to EVs produced by cells grown in conventional 2D cultures [171].

Stirred-tank bioreactors can be used as a scalable option that provides homogenization of the cell culture medium through mechanical stirring. Microcarrier technology can be used in combination with these bioreactors for expanding adherent cells and providing a high surface-area-to-volume ratio [166]. Similar to hollow-fibers, stirred-tank bioreactors can enhance EV productivity as high as 100-fold in comparison to conventional 2D culture flasks [172]. 

In the context of scalable platforms available for EV production, our group recently employed a single-use Vertical-Wheel^TM^ bioreactor for the production of human MSC-EVs [173]. This system provides a gentler and more homogenous mixing in comparison to the stirred-tank configuration, while also making use of microcarriers for adherent cell culture [174]. Our work revealed an average yield improvement of 6-fold referred to the final EV concentration in the culture medium, in comparison to culture flasks [173]. 

The use of different culture media during conditioning periods for EV obtention also impacts EV productivity and can potentially render the process more efficient. For instance, the use of non-supplemented OptiMEM (i.e., optimized formulation of Eagle’s Minimal Essential Medium (MEM)) led to an increase in the production of HEK293T-derived EVs, in comparison to OptiMEM supplemented with 10% fetal bovine serum (FBS), DMEM (i.e., Dulbecco’s Modified Eagle’s Medium) supplemented with 10% FBS, or DMEM supplemented with 10% EV-depleted FBS, among others [175]. 

In order to circumvent the low yields associated with EV production from cell culture supernatants (i.e., conditioned media), the production of EV-like particles has also been employed. This is achieved through the complete disruption of the cellular membrane leading to the release of self-assembled particles, reaching production yields that can be as high as 100-fold compared to normal EV release [176]. The most common approach to achieve this is extrusion, whereby cells are filtered through sequentially smaller pores [168].

Growing adherent cells as 3D cell aggregates instead of monolayers is another viable option to increase EV production yields. The formation of 3D MSC aggregates provides a microenvironment that better mimics in vivo conditions leading to the maintenance of MSC phenotype and innate MSC properties, which could explain the higher yields attained with this strategy. Moreover, 3D scaffolds into which cells can adhere and proliferate can also be used to promote the production of EVs and even to enhance their functional activity. This was demonstrated using bone marrow-derived MSC seeded into collagen scaffolds, which produced 2-fold more EVs compared to 2D culture conditions and enhanced the regenerative capacity of these EVs in traumatic brain injury mouse models [177].

Importantly, clinical trials employing bioreactors for EV production are already ongoing. Recently, Codiak Biosciences developed exoSTING, a therapeutic EV-based product in which EVs are loaded with stimulators of the interferon gene for the treatment of multiple cancers [178]. This strategy is currently under clinical testing in a phase 1/2 trial (NCT04592484).

Of note, there is still a gap in our knowledge concerning how the upstream conditions influence the characteristics and the quantity of produced EVs. For instance, in the context of the manufacturing of monoclonal antibodies, the optimization of upstream conditions was able to lead to improvements of 10–100-fold in product titers over the years [166]. Therefore, by adjusting operational parameters in the process such as the composition of culture media, oxygen concentration, materials used, as well as the cell passage, level of confluency, and viability, one can expect substantial improvements in EV yields.

### 5.2. Downstream Processing

The downstream processing of EVs, which comprises all the steps following upstream processing to recover and isolate EVs from cell culture conditioned medium and eventually purify them, also deserves special attention considering the particular characteristics of EVs. Over the last years, the emergence of EVs as promising tools for the development of new therapeutic options, as well as valuable structures to understand organismal pathophysiological mechanisms, has prompted the development of EV isolation methods.

However, currently available isolation methods still present considerable limitations, including poor standardization, the alteration of physicochemical properties of the isolated EV population, and the modification of EV cargo profiles. For instance, the miRNA profile in exosomes obtained from blood serum was reported to be different depending on the isolation method used, when comparing a precipitation-based isolation method with ultracentrifugation [179]. Likewise, the purity of EV samples largely depends on the isolation method used, and a compromise between the purity of a given sample and the amount of recovered EVs has to be made [180,181].

Isolation methods available can be categorized based on the principle used for the isolation of EVs from other particles present in a body fluid or cell culture conditioned medium. These methods can be generally divided into ultracentrifugation-based methods, precipitation, size-based, and microfluidics, among others.

#### 5.2.1. Ultracentrifugation-Based Methods

Ultracentrifugation methods can be divided into two distinct techniques: differential ultracentrifugation, and density gradient centrifugation. Differential ultracentrifugation is based on differences in the size and density of particles. It subjects the samples to multiple low-speed centrifugations to remove dead cells and debris, which correspond to the larger biological particles present in the sample. The low centrifugation speed periods are followed by high-speed centrifugations of 100,000× *g* (or more) in order to pellet the smaller EVs and allow the collection of these particles [182,183]. Differential ultracentrifugation is the most employed primary method of EV isolation, with more than 75% of participants reporting its use in a worldwide survey performed by the International Society for Extracellular Vesicles (ISEV) in 2020 [184]. It can be considered a method with intermediate recovery and specificity [185] and is capable of processing large volumes of fluid [186]. However, it is highly laborious and time-consuming, presenting the possibility of causing damage to EV structure, which can affect their performance for a specific application [183]. This method also presents low reproducibility and may lead to protein aggregation [182,183]. Nonetheless, numerous pathological conditions were already shown to benefit from interventions based on the administration of MSC-EVs isolated through differential ultracentrifugation. These include bone disorders [187], skeletal muscle [188] and spinal cord injuries [189,190], as well as cardiac [191] and liver [192] pathologies. 

Density gradient ultracentrifugation is a low recovery, high specificity method [185] that consists in the ultracentrifugation of particles in a biocompatible medium, sucrose, or iodixanol, with a gradient of concentrations that allows the isolation of EVs based on their density [182]. Although this method provides higher purities than differential ultracentrifugation [182,183], it is also time-consuming and the duration and force of the centrifugation can affect the quality of EVs. In addition, the media used may negatively impact the therapeutic activity of the isolated EVs [193]. Furthermore, this method is largely instrument-dependent, and residual contaminants are often co-isolated with EVs, requiring highly trained technicians to be performed well [182,183].

#### 5.2.2. Precipitation-Based Methods

Precipitation-based methods aim to lower the hydration levels of EVs using polymers, such as polyethylene glycol (PEG), or other synthetic hydrophilic polymers prone to interact with water molecules [183]. This is a high recovery and cost-effective method that is instrument-independent and can be used for large samples [182]. This method uses lower-speed centrifugation cycles in comparison to differential ultracentrifugation [183], thus not being as mechanically harsh. In fact, precipitation-based methods, in opposition to ultracentrifugation techniques, may preserve the proteins naturally bound to the surface of EVs, which is important for the downstream analysis of EV function [194]. However, this approach lacks specificity, providing a high contamination of the sample and leading to the precipitation of non-vesicular components, such as lipids, nucleic acids, and proteins [183]. Additionally, the precipitation reagents also influence the biological activity of the isolated EVs [195].

#### 5.2.3. Size-Based Methods

Size-based methods consist mainly of filtration-based separation methods and size exclusion chromatography (SEC). Ultrafiltration uses a filter membrane with a defined size exclusion limit to filtrate the particles present in a sample. It is less costly and faster than ultracentrifugation, and it is easy to operate presenting good portability, and providing high purity of isolated particles [183]. MSC-EVs isolated through this method have shown angiogenic stimulatory activity [196], a property that can be harnessed for the treatment of a wide spectrum of conditions, as well as therapeutic activity in other diseases such as osteoarthritis [197]. However, low recovery, membrane clogging and EV trapping are disadvantages usually associated to this method [182]. Tangential flow filtration (TFF) is another filtration-based technique that attenuates the membrane clogging faced in ultrafiltration due to the tangential flow of fluid across the membrane surface instead of cross-flow. TFF is more efficient and gentler than differential centrifugation, providing a higher recovery and the isolation of fewer single macromolecules and aggregates [198]. Additionally, it allows the processing of large volumes of fluid and is a time-efficient, scalable, reproducible, and robust technique [198]. This method has already been used to isolate human adipose tissue MSC-EVs, that upon intra-articular injection attenuated osteoarthritis progression and prevented cartilage degeneration in mouse models [199]. Additionally, MSC-EVs isolated through TFF displayed the ability to improve alveolarization and vascularization in animal models of bronchopulmonary dysplasia, reducing the damage induced by hypoxia [200]. This further demonstrates that EVs isolated through TFF can maintain their biological therapeutic activity.

Size exclusion chromatography (SEC) allows the separation of particles based on their interaction with the pores present in the stationary phase. Sample particles with higher dimensions will not be retained within such pores and, as a result, will be eluted first (i.e., before smaller particles). This represents a gentle separation method of EVs that maintains the structural integrity and biological activity of the vesicles, which can be illustrated by the fact that SEC isolated MSC-EVs, but not ultracentrifugation isolated MSC-EVs, maintained their immunomodulatory activity, impairing T cell proliferation [201]. Moreover, this technique is easy to operate and provides a higher purity than differential ultracentrifugation [183]. Despite these advantages, SEC may suffer from pore blockage, it is a time-consuming method, and presents high equipment costs. Additionally, SEC involves sample dilution, thus usually requiring a concentration step afterward, which can further impose yield losses on the process.

#### 5.2.4. Microfluidic-Based Methods

Microfluidic-based methods utilize microscale devices to isolate EVs. These methods can be based on EV-specific surface markers, such as microfluidic-based immunoaffinity, which rely on surfaces coated with antibodies for isolation. For example, a microfluidic device functionalized with antibodies against the tetraspanin protein, CD63, was able to efficiently isolate circulating EVs, being well suited for exosome-based diagnostics [202]. Beads coated with capture antibodies can also be employed, allowing to obtain higher EV yields [203]. In the future, this methodology may even allow isolating MSC-EV subpopulations showing increased therapeutic effects; however, the identification of such subsets is still challenging [193]. Microfluidic devices for EV isolation can also be based on EV size and density, including approaches such as membrane filtration, nano-wire-based traps, nano-sized deterministic lateral displacement, and acoustic isolations [204]. Overall, these methods are simple and efficient, as well as easy to automate, and they provide higher sensitivity than differential centrifugation. 

#### 5.2.5. Other Methods

Other relevant methods based on different EV properties are available. For instance, anion exchange chromatography is a method that takes advantage of the negative surface charge of EVs for their isolation. This method presents a similar yield, morphology, and size in comparison to differential ultracentrifugation [205]. MSC-EVs purified through this technique demonstrated the ability to prevent the onset of type 1 diabetes and uveoretinitis [206], showing the maintenance of the biological activity of the isolated MSC-EVs. Nevertheless, anion exchange chromatography may co-isolate particles with a negative net charge such as proteins. Thus, combining it with another technique such as SEC may provide greater specificity.

Apart from the microfluidic approach to the immunoaffinity isolation principle, other approaches based on chromatography are available. This method is based on the interaction of the EV surface proteins, such as CD9 or CD63 with antibodies present in the chromatography column. This provides a high sensitivity and high specificity towards specific EV subpopulations. Additionally, it is a gentle approach that conserves EV morphology [183]. Nevertheless, this is associated with low EV recovery and high costs, not being suitable for processing large volumes [183].

#### 5.2.6. Combination of EV Isolation Methods

More than 45% of researchers included in a worldwide ISEV survey in 2020 reported the use of not a single method for the isolation of EVs, but a combination of techniques [184]. This approach is particularly beneficial because it allows obtaining higher purities than single isolation methods, and certain combinations of methods were already applied in studies with different applications for EVs.

TFF combined with SEC is becoming the preferred approach, in comparison to the typically used ultracentrifugation. TFF and SEC, which feature easy scalability and are able to comply with good manufacturing practices (GMP), were already studied in the context of an EV-based strategy that intends to utilize HEK293-derived engineered EVs carrying an immunostimulatory cytokine fusion protein, the heterodimeric IL-15/lactadherin, for a targeted cancer immunotherapy approach [207]. Importantly, such a strategy can be combined with bioreactor cultivation at upstream as a scalable and efficient method to produce purified bioactive EVs, which has the potential to be translated into industrial EV production, allowing the processing of large volumes (in the scale of liters of conditioned medium), without significant changes in size and morphology. A combined TFF and SEC approach was already employed in the isolation of MDA-MB-231-derived EVs to demonstrate the function of an innovative platform that permits the study of EV-mediated RNA delivery, the CRISPR-operated stoplight system for functional intercellular RNA exchange [208]. Additionally, SEC may also be combined with ultracentrifugation to improve the separation of EVs from lipoproteins, providing an increased purity [209]. 

Currently, in the field of EVs, there is still no isolation method that can provide a high recovery of EVs combined with a high specificity [185]. As such, a compromise is always required between these two variables when choosing a suitable downstream strategy. This factor, combined with overall unsatisfactory efficiencies and high associated costs, are still the main bottlenecks that undermine large-scale EV production [183]. Other factors, such as the type of biological raw materials from where EVs are isolated, the starting volume to be processed, as well as the quantity of EVs desired and their attributes, must also be considered when choosing an isolation method or a combination thereof, which should aim to be highly efficient, scalable, and GMP compliant.

## 6. Future Perspectives

**Figure 3:**
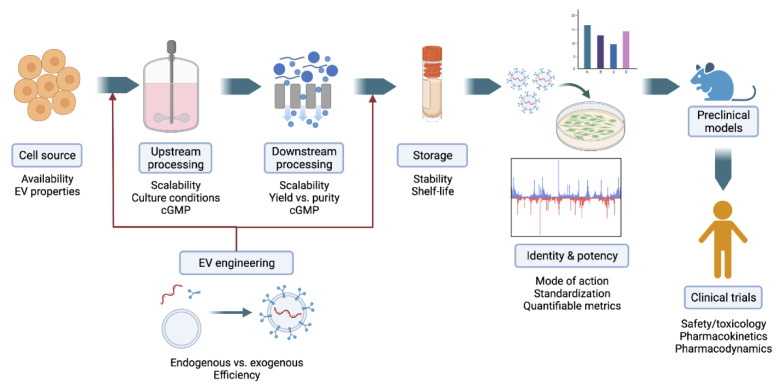
Key factors for the translation of extracellular vesicle (EV)-based therapeutics into clinical settings. Figure created with BioRender.com.

In spite of significant advances made in the field of EVs over the last decade, the translation of EV-based therapies into clinical settings still faces several challenges ahead (Figure 3). Some of these are common to the challenges faced by cell therapies (previously reviewed by our group) [210] due to the cellular origin of EVs, while others are specific to the nature of EVs.

Establishing efficient but safe and reproducible methods for EV drug loading, and to engineer EV targeting, is still challenging and will certainly continue to be the focus of further research. The methods used for exogenous EV loading show low efficiencies, while genetic modification of EV-secreting cells for vesicle modification (either for drug loading or targeting purposes) is still troublesome and difficult in primary cells. Additionally, novel strategies have been developed over the last few years aiming to modify the surface of EVs after their isolation with significant progresses, a trend expected to continue in the following years.

The selection of appropriate EV-secreting cells and the tissue of origin of the cells needs to be carefully considered since each cell type will originate EVs with different properties (e.g., proteins and RNA packaged inside them). In addition, each cell source presents different features, including the availability and easiness to isolate from human tissue sources, cell expansion ability, and EV secretion capacity.

Appropriate cell culture conditions need to be implemented in order to assure reproducibility and compliance with GMP guidelines. Scalable processes need to be implemented for EV production in order to achieve the production of high numbers of EVs required for clinical application. Bioreactors of different geometries and configurations offer several options in order to achieve this goal, as reviewed in Section 5.1. The use of serum-/xeno(geneic)-free (S/XF) culture conditions, will also be advantageous for clinical translation. Several cell culture platforms have been tested in pioneering studies aiming to achieve a scalable production of EVs, including hollow-fiber bioreactors [211,212] and spinner systems [172], among others. 

In the last 10 years, our group established different platforms for the scalable expansion of human MSC from different sources under S/XF conditions [213,214,215,216]. Recently, we further developed this work and established a platform for the scalable production of human MSC-EVs under S/XF conditions by combining the Vertical-Wheel^TM^ bioreactor system with a human platelet lysate culture supplement [173]. All of these studies allowed a significant improvement of EV production yields. Still, further work needs to be developed in order establish platforms able to reproducibly manufacture EVs at even larger scales and at a competitive cost, amenable to clinical translation. The optimization of bioreactor operation parameters, such as agitation, oxygen concentration, temperature, pH, and culture medium formulation, will be essential to maximize EV productivity. Thus, further work addressing the impact of these parameters on EV production is deemed necessary.

Similarly, EV isolation processes will need to assure reproducibility, GMP compliance, and scalability, whilst balancing suitable EV purity and yields, as reviewed in Section 5.2. Novel strategies with promising application for scalable EV isolation include combining SEC with bind-elute chromatography, whereby smaller contaminants penetrate beads and bind to its core, allowing EVs to flow through at high recovery yields [217]. Alternatively, asymmetric depth-filtration allows the immobilization of EVs at the surface and within the depth of a porous medium, while smaller particles are eluted, followed by EV recovery upon reversion of the carrier flow through the filter [218]. In the future, EV manufacturing could even benefit from integrated production and isolation processes under continuous operation in order to improve productivity and reduce costs, similar to other biopharmaceutical products (e.g., monoclonal antibodies) [219,220,221,222].

Envisaging their use as off-the-shelf products, appropriate storage conditions for EV products must be clearly defined and their stability must be assured. Strategies may be implemented in order to prolong the stability and shelf-life of EVs, such as the addition of trehalose and human albumin to EV suspensions. Trehalose, in particular, is a natural sugar that stabilizes proteins, cell membranes, and liposomes, decreases intracellular ice formation during freezing and prevents protein aggregation, being widely used in the food and drug industry, which recently revealed the capacity to prevent aggregation and cryodamage of EVs [223,224], as well as improving the short-term and long-term storage of EVs when combined with human albumin [225]. Nevertheless, only a few studies to date have addressed the storage of EV products. Further work will be required, studying EV stability and potency after storage, in conditions mimicking potentially subsequent clinical trials, in order to appropriately translate pre-clinical studies into the clinical scenario.

The translation of EV products to clinical practice will require establishing standardized identity criteria and potency assays for EVs, allowing cross comparison between different laboratories, thereby supporting the development and validation of EV-based therapies and their progression to clinical testing. Motivated by this need, a consortium of researchers recently established identity criteria including quantifiable metrics for MSC-EVs [226], and also presented requirements for the development of standardized potency tests for the therapeutic application of these EVs [227]. It is important to clearly define the mechanisms of action of EV-based therapeutics for clinical translation, and these should be reflected in suitable potency assays. However, a full elucidation of the therapeutic mechanism of EVs is challenging, since it will be multifaceted and vary between disease models.

Afterwards, appropriate preclinical models must be selected to characterize the safety and toxicology of therapeutic EVs, as well as their pharmacokinetic and pharmacodynamic profiles [109]. Information from these studies will be relevant to determine proper doses for clinical studies, which will be challenging given the current heterogeneity of EV preparations and their different therapeutic potency, depending on the targeted disease. Importantly, pre-clinical studies and current clinical trials indicate that EVs are generally safe and well tolerated [13,14,15,16,128].

To conclude, there is a long road ahead for the application of EV therapeutics in the clinical setting, as the field of EVs is still at its infancy. Nevertheless, it is already clear that EVs will likely give rise to relevant new therapeutic solutions, given their unique set of characteristics compared with synthetic nanocarriers, as well as chemical and biological products, including cell therapies.

## Figures and Tables

**Table 1:** Overview of strategies to engineer targeting of extracellular vesicles (EVs) through genetic modification of producing cells to express a targeting moiety fused to an EV transmembrane protein.

EV Transmembrane Protein	Targeting Moiety	Target	Purpose	Refs.
Lamp2b	RVG peptide	Acetylcholine receptor in neurons	Deliver BACE1 siRNA for Alzheimer’s disease treatment	[7,149]
	RVG peptide	Acetylcholine receptor in neurons	Deliver miR-124 to promote neurogenesis after stroke	[153]
	iRGD peptide	α_v_ integrin-positive breast cancer cells	Deliver doxorubicin	[136]
	IL3 fragment	IL3 receptor in CML cells	Deliver Imatinib or BCR-ABL siRNA	[151]
	Cardiomyocyte specific peptide	Cardiomyocytes	Target cardiomyocytes	[154]
PDGFR	GE11 peptide	EGFR-expressing cancer cells	Deliver let-7a miRNA	[155]
GPI-anchoring peptide	Anti-EGFR nanobody	EGFR-expressing cancer cells	Target cancer cells	[152]

Lamp2b—lysosome-associated membrane protein 2; RVG—rabies viral glycoprotein; BACE1—beta secretase 1; IL3—interleukin 3; CML—chronic myeloid leukemia; PDGFR—platelet-derived growth factor receptor; EGFR—epidermal growth factor receptor; GPI—glycosylphosphatidylinositol.

**Table 2:** Overview of strategies to engineer extracellular vesicles (EVs) targeting by anchoring a targeting moiety to EVs after its production and isolation.

Linkage Method	Targeting Moiety	Target	Purpose	Refs.
Post-insertion of phospholipid (DMPE)-PEG fusion molecules in EV membranes	Anti-EGFR nanobody (conj. with DMPE-PEG)	EGFR-expressing cancer cells	Target cancer cells	[158]
Membrane anchoring cholesterol	AS1411 DNA aptamer (conj. with cholesterol)	Nucleolin on breast cancer cells	Deliver let-7 miRNA or VEGF siRNA	[159]
Electrostatic interaction between cationized pullulan and EVs	Cationized pullulan (a polysaccharide polymer)	Hepatocyte asialoglycoprotein receptors	Target injured liver	[160]
C1C2 domain of lactadherin binding to PS present on EV membrane	Anti-EGFR nanobody (conj. with C1C2)	EGFR-expressing cancer cells	Target cancer cells	[156]
Membrane anchoring cholesterol	RNA aptamers or folate (conj. with cholesterol)	PSMA, EGFR or folate receptor on prostate, breast, or colorectal cancers, respectively	Deliver survivin-targeting siRNA	[161]
Click chemistry reaction	c(RGDyK) peptide	Integrin α_v_β_3_ in reactive cerebral vascular endothelial cells after ischemia	Deliver curcumin to stroke lesions	[162]
ApoA-I mimetic peptide interaction with phospholipids on EV membrane	LDL peptide	LDL receptor on GBM cells	Delivery of KLA peptide and methotrexate	[163]
CP05 peptide binding to CD63 present on EV membrane	Muscle targeting peptide M12 (conj. with CP05)	Muscle	Deliver PMO to muscle for Duchenne muscular dystrophy treatment	[164]
Click chemistry reaction	NRP-1 targeting peptide (RGE)	NRP-1 in glioma cells and tumor vascular endothelium	Deliver SPIONs and curcumin for imaging and therapy of glioma	[157]
Covalent bond by protein ligating enzymes Sortase A or OaAEP1 ligase	EGFR-targeting peptide or nanobodies targeting EGFR or HER2	Cancer cells expressing EGFR or HER2	Deliver paclitaxel or mRNA	[165]

DMPE—1,2-Dimyristoyl-sn-glycero-3-phosphoethanolamine; PEG—polyethylene glycol; EGFR—epidermal growth factor receptor; VEGF—vascular endothelial growth factor; PS—phosphatidylserine; PSMA—Prostate-specific membrane antigen; LDL—low-density lipoprotein; GBM—glioblastoma multiforme; PMO—phosphorodiamidate morpholino oligomer; NRP-1—neuropilin-1; SPION—superparamagnetic iron oxide nanoparticles; HER2—human epidermal growth factor receptor 2.

## Data Availability

No new data were created or analyzed in this study. Data sharing is not applicable to this article.
